# Association of Multiple Indicators of Pubertal Timing with Depressive Symptoms and Depression in Adolescent Girls

**DOI:** 10.1192/bjp.2025.88

**Published:** 2025-08-26

**Authors:** Dana Tarif, Jon Heron, Abigail Fraser, Ahmed Elhakeem, Carol Joinson

**Affiliations:** 1 Department of Population Health Sciences, University of Bristol, Bristol, UK

**Keywords:** Pubertal Timing, Depression, ALSAPC, Adolescence

## Abstract

**Background:**

Previous studies investigating the association between pubertal timing and depression in girls primarily use self-reported age at menarche (AAM). This study examines associations of a wide range of pubertal timing indicators, including anthropometric and self-reported measures, with depressive symptoms and depression during adolescence and early adulthood.

**Aims:**

Compare associations of multiple indicators of pubertal timing with depressive symptoms and depression in girls and explore whether these associations persist into early adulthood.

**Methods:**

The study sample comprised 4,607 girls from the UK-based Avon Longitudinal Study of Parents and Children (ALSPAC). Seven measures of pubertal timing were assessed between ages 7 to 17 [age at: peak height velocity (aPHV), peak weight velocity; peak bone mineral content velocity; age at Tanner pubic hair and breast development stage 3; age at axillary hair, and AAM]. Depressive symptoms were measured at 14, 17, 18 and 24 years using the Short Mood and Feelings Questionnaire. Depression was assessed at 15, 18 and 24 years using the developmental and wellbeing assessment and the Clinical Interview Schedule-revised. Multivariable logistic regression models were adjusted for socioeconomic status and pre-pubertal body mass index.

**Results:**

Later pubertal timing was associated with lower odds of depressive symptoms at age 14 across six measures, including aPHV (AOR: 0.82; 95% CI 0.72-0.95) and AAM (AOR: 0.84; 95% CI 0.76-0.92). Later AAM and Tanner breast stage 3 were associated with lower odds of depression at age 18 (AOR: 0.85; 95% CI 0.75, 0.97 and AOR: 0.83; 95% 0.72, 0.95, respectively). These associations attenuated by age 24.

**Conclusions:**

Later pubertal timing, measured through objective and self-reported indicators, was associated with reduced odds of depressive symptoms during mid-adolescence, with associations attenuating by early adulthood. Inconsistent associations of some self-reported measures (e.g. axillary hair and Tanner pubic hair) suggests a need for caution in their interpretation.

## Introduction

There is robust evidence for an association between early pubertal timing in girls and depressive symptoms (1–3), however, there continues to be substantial variation in the measurement of pubertal timing in the literature (4, 5). As puberty is a developmental process and not a fixed event, its measurement can pose a methodological challenge. While age at menarche (AAM) is the most frequently used measure of pubertal timing, self-reported indicators such as the Tanner stages of development are widely used (6, 7). These measures are subject to potential misreporting and can be influenced by embarrassment or cultural limitations, particularly when illustrations that depict only Caucasian adolescents are used (8). Despite these limitations, self-reported indicators show good agreement with objective measures of pubertal timing, such as age at peak height velocity (aPHV), age at peak weight velocity and age at peak bone mineral content (BMC) velocity, which have been used to explore associations between puberty and mental health (9–12). These measures can be derived from longitudinal anthropometric data using parametric growth curve modes, such as Superimposition by Translation and Rotation (SITAR) analyses, which estimate the peak velocity of change during the pubertal growth spurt (13, 14). Growth curve analysis can be applied to evaluate timing of secondary sexual characteristics, such as Tanner stages and axillary hair (13). This study, based on data from a large UK birth cohort, compares associations between a range of pubertal timing indicators—both objective and self-reported—with depressive symptoms and depression, and explores whether these associations persist into early adulthood. The primary analysis explores depressive symptoms in mid-adolescence, where there is a high degree of inter-individual variation in pubertal maturation, and depressive symptoms/depression diagnosis in late adolescence/early adulthood, where most girls will have gone through the pubertal transition.

## Methods

### Participants

The Avon Longitudinal Study of Parents and Children (ALSPAC) is a birth cohort that originally recruited pregnant women (n = 14,541) residing in Avon, UK with expected dates of delivery 1st April 1991 to 31st December 1992. Of the initial pregnancies, there were 14,676 foetuses, 14,062 were live births and 13,988 children who were alive at 1 year of age. When the oldest children were approximately 7 years of age, an attempt was made to increase the original sample by recruiting eligible individuals who did not join the study. This resulted in a total sample size of 15,454 pregnancies (15,658 foetuses, 14,901 alive at 1 year of age) when using data after the age of seven. Due to confidentiality reasons, data on 13 triplets/quads is not provided resulting in 15,645 cases (15–17). Of these, 50.8% were female (assigned sex at birth), resulting in a sample of 7,961 (for participant flow chart, see Supplementary Material S1).

The authors assert that all procedures contributing to this work comply with the ethical standards of the relevant national and institutional committees on human experimentation and with the Helskinki Declaration of 1975 as revised in 2013. All procedures involving human subjects/patients were approved by ALSPAC Law and Ethics Committee and Local Research Ethics Committees. Informed consent for the use of data collected via questionnaires and clinics was obtained from participants following the recommendations of the ALSPAC Ethics and Law Committee at the time (http://www.bristol.ac.uk/alspac/researchers/research-ethics/). Study data were collected and managed using REDCap electronic data capture tools hosted at the University of Bristol (18). REDCap (Research Electronic Data Capture) is a secure, web-based software platform designed to support data capture for research studies. Please note that the study website contains details of all the data that is available through a fully searchable data dictionary and variable search tool (http://www.bristol.ac.uk/alspac/researchers/our-data/).

### Measures

#### Pubertal timing measures

Data on seven indicators of pubertal development were collected from 7 to 17 years. These included: age at peak height velocity (aPHV), age at peak weight velocity (aPWV), age at peak BMC velocity (aPBMCV), age at Tanner pubic hair stage 3, age at Tanner breast development stage 3, age at axillary hair (‘started growing hair’) and age at menarche (AAM). At research clinics, data on height and weight were collected by trained fieldworkers and data on BMC (total body less head) were collected using dual-energy X-ray absorptiometry (DXA) scans. Self-reported Tanner stages, menarche and axillary hair were collected in questionnaires. aPHV, aPWV and aPBMCV, age at Tanner pubic hair stage 3, age at Tanner breast development stage 3 and age at axillary hair were derived using Superimposition by Translation and Rotation (SITAR) analysis (14), described in detail elsewhere (10). SITAR is a nonlinear mixed effects model that fits a mean growth curve to a study sample and uses random effects to describe individual variations in size, timing and intensity. This model is particularly well-suited for capturing heterogeneity in changes that typically progresses monotonically but follow a nonlinear pattern, such as growth or pubertal development. AAM was obtained from the first reported age at onset of menstruation from clinic reports and postal questionnaires. For distribution of pubertal timing measures, see Supplementary Material S2.

*Depressive Symptoms* were measured at 13.84 (SD = 0.21) and 17.84 (SD = 0.40) years old, hereafter referred to as 14 and 18 years, using the Short Moods and Feeling Questionnaire (SMFQ) (19), a widely used and validated tool for depressive symptoms in adolescents (20). The SMFQ is a 13-item questionnaire measuring the occurrence of depressive symptoms over the past 2 weeks. The total scores on the questionnaire range from 0-26, with higher scores indicating greater depressive symptoms. Due to positively skewed distribution of the SMFQ responses, we used SMFQ scores of 11 and greater to indicate high levels of depressive symptoms, because this threshold has previously been found to have good specificity for predicting clinical depression (20, 21).

*Depression* was measured at 17.82 (SD = 0.46) years old, hereafter referred to as 18 years, using a computerised version of the Revised Clinical Interview Schedule (CIS-R) (22). The CIS-R is a widely used and validated self-report questionnaire that assesses depression in community samples (22). The CIS-R asks about a range of symptoms experienced over the past week that are used to generate a depression diagnosis according to the International Classification of Diseases (ICD-10) criteria (23). The outcome used in this study was any ICD-10 diagnosis of depression (mild, moderate or severe) versus none.

### Confounders

Socioeconomic status (SES) was measured by occupational social class (manual vs. non-manual) during the antenatal period, home ownership status (renter versus owned/privately rented) assessed when the study child was 1.8 years, maternal educational attainment (Certificate of Secondary Education [CSE]/Vocational qualifications/none; O-Levels; A-Levels and above) assessed when the study child was 5.1 years, major financial problems (experienced in first 5 years of child’s life versus. none) and father absence (before age 5, between age 5-10 or father present). Body mass index (BMI), measured at age 9 years, was calculated based on height and weight measurements obtained from research clinics (90.2%) and parent reported height and weight provided in questionnaires at age 9 when clinic data were missing (9.8%). Confounder selection was guided by empirical research on factors related to both pubertal timing and depression (2, 24).

### Primary Analysis

Our primary aim was to examine associations between the seven pubertal timing indices and depressive symptoms in mid-adolescence and depressive symptoms/depression diagnosis in late adolescence/early adulthood. For this analysis, we used three outcomes – depressive symptoms (SMFQ) data at ages 14 and 18 years, and depression diagnosis data (CIS-R) at age 18. We used logistic regression analysis, adjusted for the confounders. In a preliminary analysis, age at response was adjusted for to account for slight variations in participants’ age at outcome assessment. This had no meaningful impact on the results and was therefore not included in the main analysis. The potential for non-linear relationships was examined using quadratic and fractional polynomial regressions and we found no evidence of departure from linearity. All analyses were carried out using Stata 17 (25).

### Post-hoc Analysis

Based on the results from the primary analysis, we conducted post hoc analyses with additional timepoints to examine if associations between the pubertal timing indices and depressive symptoms/depression were consistent over time. For these analyses, we used four additional outcome variables available in the ALSPAC dataset. Depressive symptoms were measured using the SMFQ at 16.67 (SD = 0.23) and 23.87 (SD = 0.52) years old, hereafter referred to as 17 and 24 years. Depression was measured using the CIS-R at 24.45 (SD = 0.82) years old, hereafter referred to as 24 years. While there were no earlier timepoints for the CIS-R, depression was measured using the developmental and wellbeing assessment (DAWBA) (26) band predictions at 15.49 (SD = 0.38) years old, hereafter referred to as 15 years. The DAWBA is designed to generate DSM-IV diagnoses for young people aged 2 to 17 years old (27). The DAWBA was self-reported via computerised questionnaire by the child at a research clinic, generating the following probability bands: “<0.1%”, “∼0.5%”, “∼15%”, “∼50%”, “>70%”. We used a probability of 50% or greater to indicate depression, a binary distinction which has been previously validated (27). We used logistic regression analysis, adjusted for the confounders.

### Missing Data

4,607 participants had at least one puberty measure available and were eligible for inclusion in this study. Complete data on the puberty variables, depression outcomes and confounders were available for 1,200 participants. Missing data were imputed using Multiple Imputation by Chained Equations (MICE) (28). 50 datasets were imputed (25 iterations), with parameter estimated pooled according to Rubin’s rules (29). The imputation model included all variables in the analyses and relevant auxiliary variables (Supplementary Material, S7).

## Results

**Figure 1:**
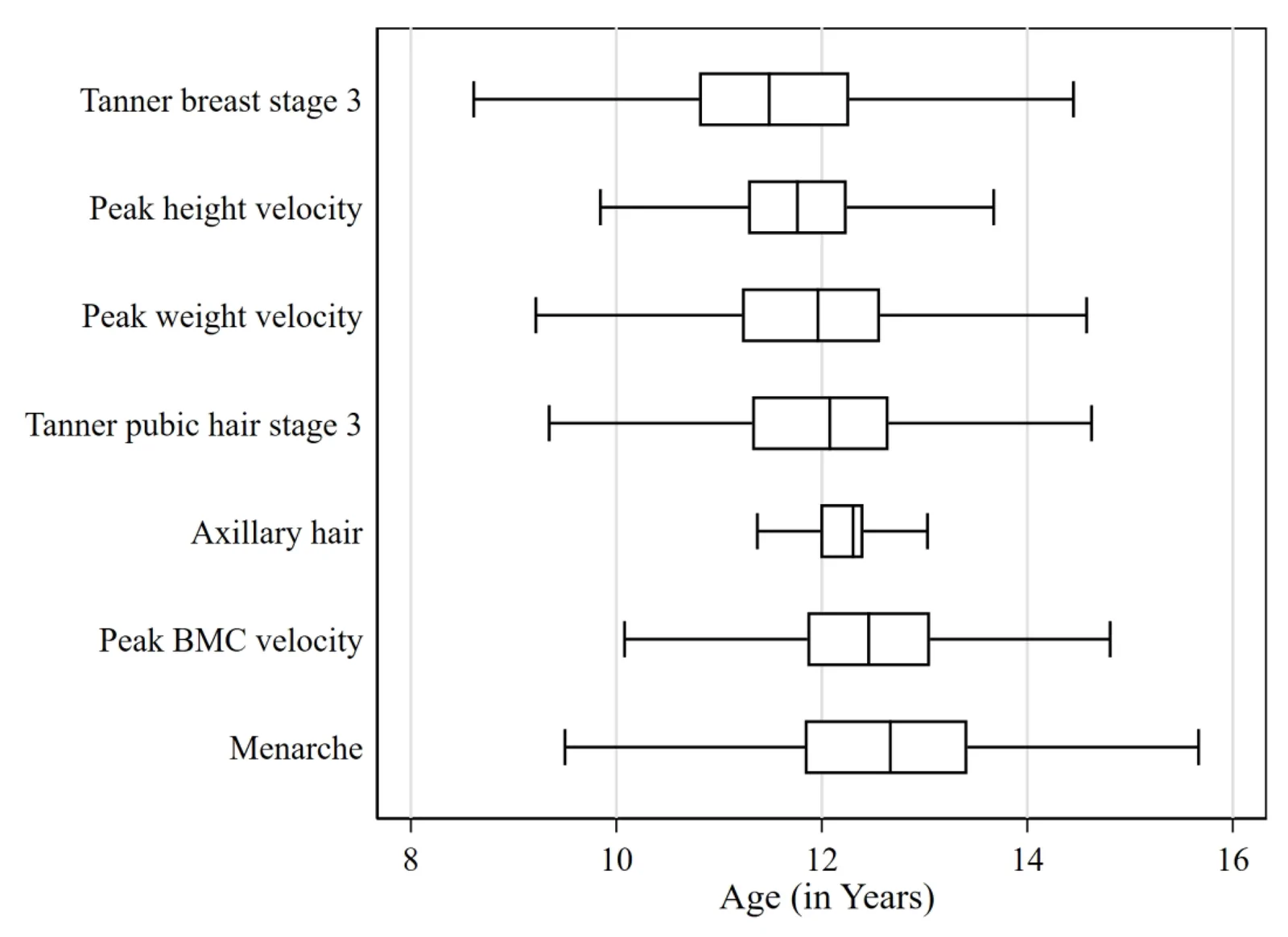
Timing of pubertal development for each pubertal timing measure in imputed sample (N=4,607).

**Figure 2:**
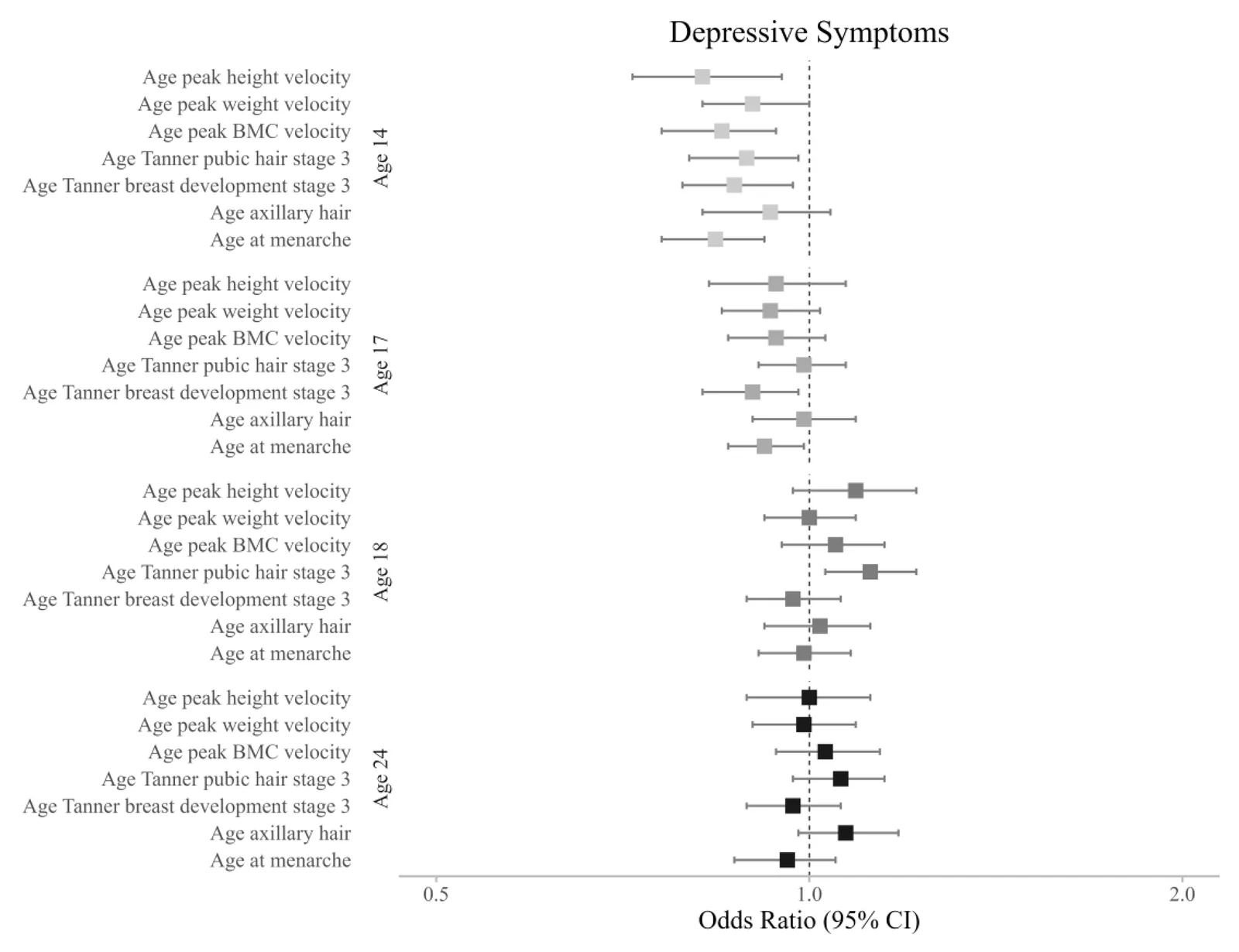
Association between pubertal timing (one-year increase) and depressive symptoms (SMFQ >=11) at 14, 17, 18 and 24 years, adjusted for SES and pre-pubertal BMI, in imputed sample (N=4,607).

**Figure 3:**
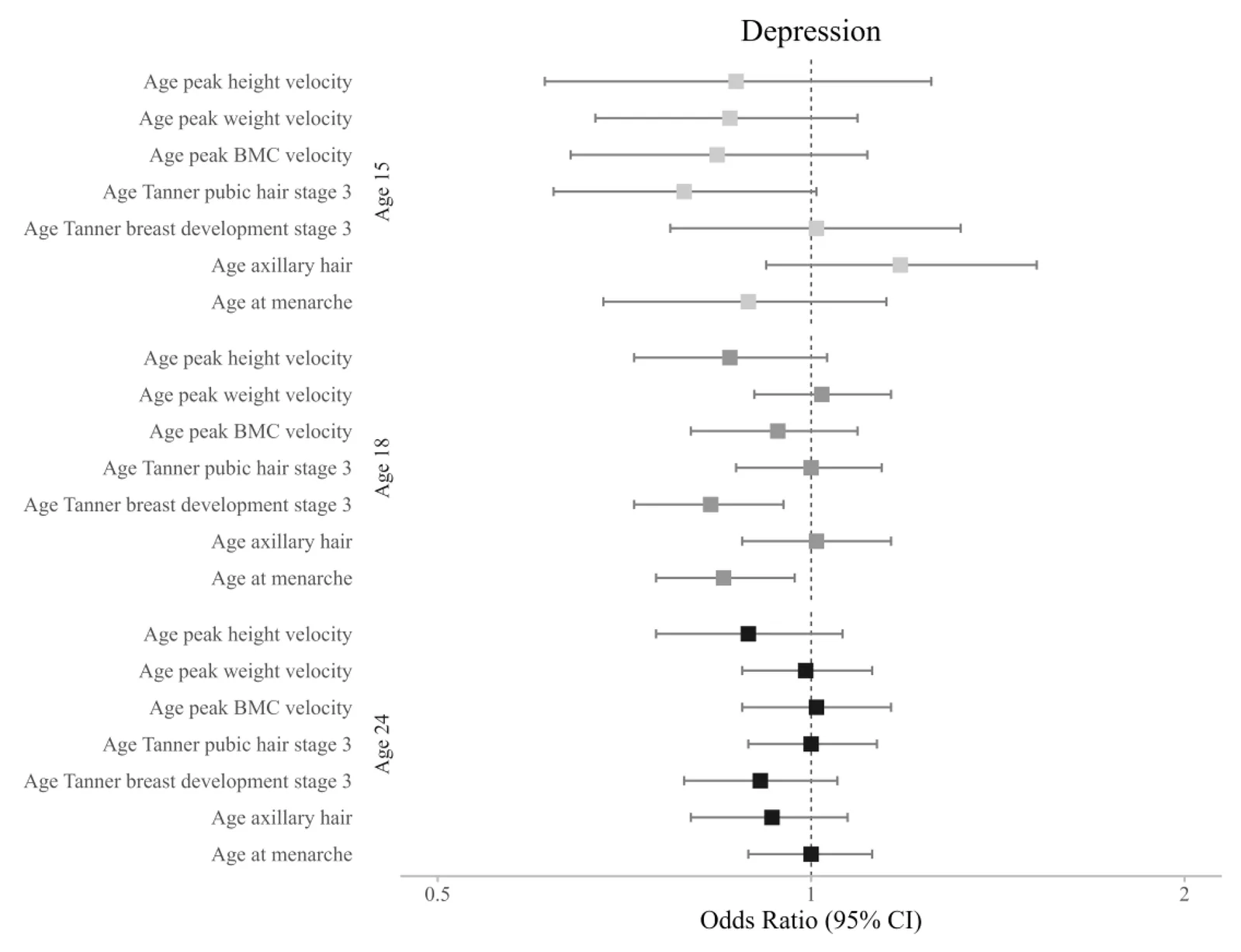
Association between pubertal timing (one-year increase) and depression at 15, 18 and 24 years, adjusted for SES and pre-pubertal BMI, in imputed sample (N=4,607).

Mean age at puberty varied from 11.8 years for Tanner breast development stage 3 to 12.7 years for age at menarche (Figure 1). Descriptive data for all puberty measures, depression outcomes and confounders are shown in Table 1. In Figures 2 and 3, results from the primary analysis and post hoc analysis are combined in order to show the pattern of associations at the different timepoints. Figure 2 shows the adjusted associations between the seven pubertal timing indicators and depressive symptoms (SMFQ>=11) at 14, 17, 18 and 24 years. Figure 3 shows the adjusted associations between the seven pubertal timing indices and depression at 15, 18 and 24 years. Unadjusted, SES-adjusted and fully adjusted results are presented in full in the Supplementary Material (S3-4), alongside the complete case results (S5-6).

### Depressive Symptoms

*Age 14*. A one-year increase in aPHV, aPWV and aPBMCV was associated with a decrease in the odds of depressive symptoms (AOR: 0.82; 95% CI 0.72-0.95, AOR: 0.90; 95% CI 0.82-1.00 and AOR: 0.85; 95% CI 0.76, 0.94, respectively). Age at Tanner pubic hair and breast stage 3 were associated with reduced odds of depressive symptoms (AOR: 0.89; 95% CI 0.80, 0.98 and AOR: 0.87; 95% CI 0.79, 0.97, respectively). AAM was also associated with reduced odds of depressive symptoms (AOR: 0.84; 95% CI 0.76, 0.92). There was no evidence that age at axillary hair development was associated with depressive symptoms at age 14 (AOR: 0.93; 95% CI 0.82, 1.04).

*Age 18*. We found little evidence that a later age at puberty was associated with increased odds of depressive symptoms. Contrary to the findings at age 14, a one-year increase in age at Tanner pubic hair stage 3 was associated with an increased odds of depressive symptoms (AOR: 1.12; 95% CI 1.03, 1.22). In the model adjusting only for SES and not pre-pubertal BMI, a later Tanner pubic hair stage 3 was not associated with depressive symptoms at age 18 (SES-adjusted OR: 1.07; 95% CI 0.98, 1.17). When adjusting only for SES, a one-year increase in aPWV and Tanner breast stage 3 were associated with a decrease in the odds of depressive symptoms (SES-adjusted OR: 0.91; 95% CI 0.85, 0.99 and OR: 0.91; 95% CI 0.84, 0.98, respectively), but not when adjusting for both SES and pre-pubertal BMI (AOR: 1.00; 95% CI 0.92, 1.09 and AOR: 0.97; 95% CI 0.89, 1.06, respectively). No associations were found with AAM, aPBMCV or age at axillary hair and depressive symptoms at 18.

### Depression

*Age 18*. A later age at Tanner breast stage 3 and a later AAM were associated with decreased odds of depression (AOR: 0.83; 95% 0.72, 0.95 and AOR: 0.85; 95% CI 0.75, 0.97, respectively). No associations were found between the remaining pubertal timing measures and depression at 18 years.

### Post-hoc Analyses

Based on the results of the primary analyses, we conducted post-hoc analyses using further timepoints to explore the consistency of associations between pubertal timing and depressive outcomes over time.

#### Depressive Symptoms

At age 17, a one-year increase in AAM and age at Tanner pubic hair stage 3 were associated with a decrease in the odds of depressive symptoms (AOR: 0.92; 95% CI 0.86, 0.99 and AOR: 0.90; 95% CI 0.82, 0.98, respectively). For the remaining five pubertal timing measures, there was weak evidence of an association with decreased odds of depressive symptoms at age 17, indicating imprecision in the estimate. At age 24, we found no evidence of an association between pubertal timing and depressive symptoms.

#### Depression

At age 15, there was weak evidence (p=0.067) for an association between pubic hair status and depression as measured by the DAWBA; a one-year increase in age at Tanner pubic hair stage 3 was associated with approximately 20% lower odds of depression (AOR: 0.79; 95% CI 0.62, 1.01). There was no evidence of an association between the remaining pubertal timing indices and depression at age 15. At age 24, we found no evidence of an association between pubertal timing and depression, as measured by the CIS-R.

## Discussion

### Overview of findings

Later pubertal timing was consistently associated with reduced odds of depressive symptoms at age 14 across six of the seven indicators of pubertal timing, including self-reported (Tanner stages, AAM) and objective measures (aPHV, aPWV, aPBMCV). There were no associations with axillary hair. At age 18, later AAM and age at Tanner breast stage 3 were associated with reduced odds of depression (CIS-R), however, in contrast to earlier findings at age 14, a later Tanner pubic hair stage 3 was associated with increased odds of depressive symptoms at age 18. Post hoc analyses were carried out to check for consistency of the results over time. At age 15, there was weak evidence for an association between later Tanner pubic hair stage 3 and lower odds of depression (measured by the DAWBA probability bands). At age 17, a later AAM and later age at Tanner pubic hair stage 3 were associated with reduced odds of depressive symptoms. By age 24, there was no clear evidence of an association between pubertal timing and depressive symptoms or depression. The post hoc analyses confirmed the consistency of these findings with the primary analysis.

The consistent findings in mid-adolescence across both objective and self-reported measures of pubertal timing supports the robustness of the association between early pubertal timing and depressive symptoms at this stage (2, 3, 5). The repeated anthropometric measures (e.g. aPHV) have several notable strengths; they offer reliable, non-intrusive alternatives to self-reported questionnaires. Participants might find it embarrassing to answer questions on menstruation and breast/pubic hair development (30), particularly when there are cultural differences in the discussion of these topics (31). Additionally, retrospective reports of menarche may suffer from recall error, with single-item reports recorded years after the event being particularly prone to inaccuracy (8). In contrast, longitudinal studies that regularly collect data on menarche are more likely to capture an accurate AAM, reducing the potential for recall error. aPHV shows a strong correlation with AAM (10), highlighting its potential as an alternative measure which can be applied to both sexes, expanding its applicability. Anthropometric measures provide an opportunity for cohort studies that already collect height and weight data to derive objective indicators of pubertal timing.

Inconsistencies were observed in the findings when we used self-reported age at axillary hair and age at Tanner pubic hair stage 3 as indicators of pubertal timing. Age at axillary hair was the only measure to show no association with depressive symptoms at age 14 and no association with depressive symptoms or depression at any age. In this study, axillary hair data were collected as a dichotomous response (presence vs absence), which may have led to imprecise estimations. It is also possible that the lack of association between age at axillary hair and depressive symptoms may reflect the differing hormonal pathways driving adrenarche (e.g. axillary hair growth) and gonadarche (e.g. breast development). Gonadarche, which is primarily driven by increases in estradiol, has been consistently linked to depression in females, whereas adrenarche may not be as strongly associated (32, 33). Inconsistencies were also found with Tanner pubic hair stage 3; a later age at Tanner pubic hair stage 3 was associated with reduced odds of depressive symptoms at age 14, but with increased odds of depression at age 18. Post hoc analyses were conducted to assess whether this result was anomalous or consistent over further time points. These analyses suggested that the conflicting results at age 18 were more likely due to measurement error in the self-reported scale rather than a true reversal of the observed association. Concerns of validity when utilising self-reported or parent-reported Tanner stages have been noted by previous studies (34, 35). In this study, pubic hair development was assessed using line drawings, which could lead to misclassification of Tanner stage, particularly as the reporting shifted from parent/carer to self-reports. Over 75% of the first five puberty questionnaires were completed with the help of a parent or guardian, compared to less than 15% of the final four questionnaires, with this shift occurring at age 14.

### Findings in context

Our findings align with the early maturation hypothesis (36), which suggests that a mismatch between physical development and emotional or cognitive development may increase vulnerability to depressive symptoms in early maturing girls. Early maturation may increase susceptibility to psychological distress through a combination of biological factors (e.g. hormonal effects) psychological factors (e.g. body image, self-efficacy) and social factors (e.g. unwanted attention, association with older peers resulting in risk-taking behaviour) (3). The association between later pubertal timing and lower odds of depressive symptoms is strongest in mid-adolescence, with the associations attenuating by early adulthood. This finding aligns with the attenuation hypothesis, suggesting that associations between pubertal timing and adverse mental health outcomes diminish over time, becoming negligible by the fourth decade of life (37, 38). It is notable that we observed differences in results when comparing depressive symptoms with depression diagnosis. This discrepancy may reflect true differences in the relationship depending on severity of depressive outcomes. Depression diagnoses require meeting a higher symptom threshold, which could attenuate associations observed with depressive symptoms. Alternatively, this discrepancy may be related to statistical power, as depression has a lower prevalence compared to depressive symptoms. In our study, one in four girls reported depressive symptoms (SMFQ>=11) at age 18, compared to one in ten girls who met the criteria for ICD-10 depression (according to the CIS-R). These findings highlight the importance of distinguishing between clinical depression and depressive symptoms in research. Understanding the extent to which pubertal timing is associated with clinical outcomes, as opposed to depressive symptoms alone, is critical for informing clinical practice.

### Strengths and Limitations

A key strength of our study is the use of multiple measures of pubertal timing, including both subjective and objective indicators. By employing repeated measures of each pubertal timing indicator, we minimised recall error, as the proximity of data collection to the pubertal events themselves reduces the likelihood of inaccurate reporting (8). This approach strengthens the validity and reliability of these measures compared to studies relying solely on retrospective recall. The longitudinal design enabled the assessment of associations between pubertal timing and depressive symptoms/depression in mid-adolescence and early adulthood, allowing us to examine if associations persisted over time.

Limitations of this study include the use of different diagnostic tools (DAWBA in childhood, CIS-R in adolescence), which complicates the comparability of the depression outcomes. However, rates of depression are expected to be low in childhood (39), thus a probable DAWBA-derived diagnosis of depression has been widely used and validated in adolescents (27). Another limitation is the reliance on self-reported data for some indicators, which may introduce measurement error. Additionally, the underrepresentation of ethnic minorities in the ALSPAC cohort (95% White ethnicity) limits the generalisability of our findings. Missing data is a common challenge in cohort studies which can lead to selection bias, particularly because excluded individuals tend to be more socioeconomically disadvantaged compared to the original cohort, and there is a higher prevalence of depression among individuals with a lower SES (40). Multiple imputation was used to address potential bias from attrition and missing data under the assumption that data were missing at random (MAR), meaning that, conditional on observed data, the probability of an observation being missing is independent of unobserved data. Whilst the MAR assumption is not directly testable, the inclusion of extensive auxiliary variables strengthens the plausibility of this assumption, minimising potential biases associated with missing data. These auxiliary variables included measures of socioeconomic status, maternal mental health and earlier indicators of mental health, such as previous measures of depressive symptoms. Multiple imputation has been shown to eliminate bias in MAR data regardless of the proportion of missing data (41). Finally, we acknowledge that multiple testing increases the risk of Type I error. However, as our analyses examined related exposures (pubertal timing indices) and related outcomes (depressive symptoms and depression) over multiple time points, the tests are not fully independent. Traditional methods for correcting p-values, which assume independence, may be overly conservative in this context. We provide exact p-values and 95% confidence intervals for all analyses to provide transparency and allow readers to interpret the strength of evidence for each association (42).

### Conclusions

Our study provides robust evidence that early pubertal timing is associated with increased odds of depressive symptoms in girls during mid-adolescence, with associations diminishing by early adulthood. Both self-reported and objective measures of pubertal timing can be used as reliable indicators, but our findings advise against using Tanner pubic hair stage 3 and age at axillary hair. Each pubertal timing indicator captures specific aspects of pubertal development, e.g. aPHV marks earlier and more visible physical changes, while others, such as AAM, reflect later, developmentally significant milestones. Researchers should carefully consider which pubertal indicators align with their study’s aims, taking into account reliability, validity and feasibility of each measure, and ensuring that it accurately reflects the developmental processes or psychological outcomes under investigation.

## Supplementary Material

Supplementary Material

## Figures and Tables

**Table 1:** Descriptive Statistics in Imputed sample (up to N=4,607)

	Mean (SE)
Age peak height velocity (in years)	11.8 (0.8)
Age peak weight velocity (in years)	11.9 (1.1)
Age peak BMC velocity (in years)	12.4 (1.0)
Age Tanner pubic hair stage 3 (in years)	12.0 (1.1)
Age Tanner breast development stage 3 (in years)	11.5 (1.1)
Age at menarche (in years)	12.7 (1.2)
Age axillary hair (in years)	12.3 (0.9)
BMI at 9	17.9 (3.0)
	**% (SE)**
Depressive symptoms^†^ at age 14 (SMFQ)	15.3 (0.7)
Depressive symptoms at age 17 (SMFQ)	22.4 (0.8)
Depressive symptoms at age 18 (SMFQ)	25.0 (0.9)
Depressive symptoms at age 24 (SMFQ)	26.6 (0.9)
Depression at age 15 (DAWBA)	2.1 (0.3)
Depression at age 18 (CIS-R)	10.3 (0.6)
Depression at age 24 (CIS-R)	12.6 (0.7)
Home ownership (renting/non-homeowner)	12.1 (0.5)
Maternal education	
< O-Levels (CSE/Vocational/None)	20.8 (0.7)
O-Levels	40.1 (0.8)
Major Financial Problems	26.6 (0.7)
Social Class (Manual)	17.1 (0.6)
Father absence	
< 5 years	8.5 (0.5)
Between 5-10 years	15.6 (0.6)

† Note: Depressive Symptoms = SMFQ>=11

## Data Availability

ALSPAC data access is through a system of managed open access. Information about access to ALSPAC data is given in the ALSPAC data management plan (http://www.bristol.ac.uk/alspac/researchers/data-access/documents/alspac-data-management-plan.pdf). Analysis code is available on request from the lead author.
