# Supplementary material for: Association of Multiple Indicators of Pubertal Timing with Depressive Symptoms and Depression in Adolescent Girls
Source: Br J Psychiatry. Author manuscript; Available in PMC 2025 Sep 9. (PMC7618095; doi:10.1192/bjp.2025.88)
Supplement: Supplementary Material [file EMS203826-supplement-Supplementary_Material.pdf]

Supplementary Material

S1. Participant Flow Chart 2

S2. Distribution of pubertal timing variables 3

S3. Associations between pubertal timing indicators (per one year increase) and depressive symptoms in imputed sample (N= 4,607). 4

S4. Associations between pubertal timing indicators (per one year increase) and depression in imputed sample (N= 4,607). 6

S5 Complete Case: Associations between pubertal timing indicators (per one year increase) and depressive symptoms in complete case samples 8

S6. Complete Case: Associations between pubertal timing indicators (per one year increase) and depression in complete case sample 10

S7. Coding of Variables 12

S1. Participant Flow Chart

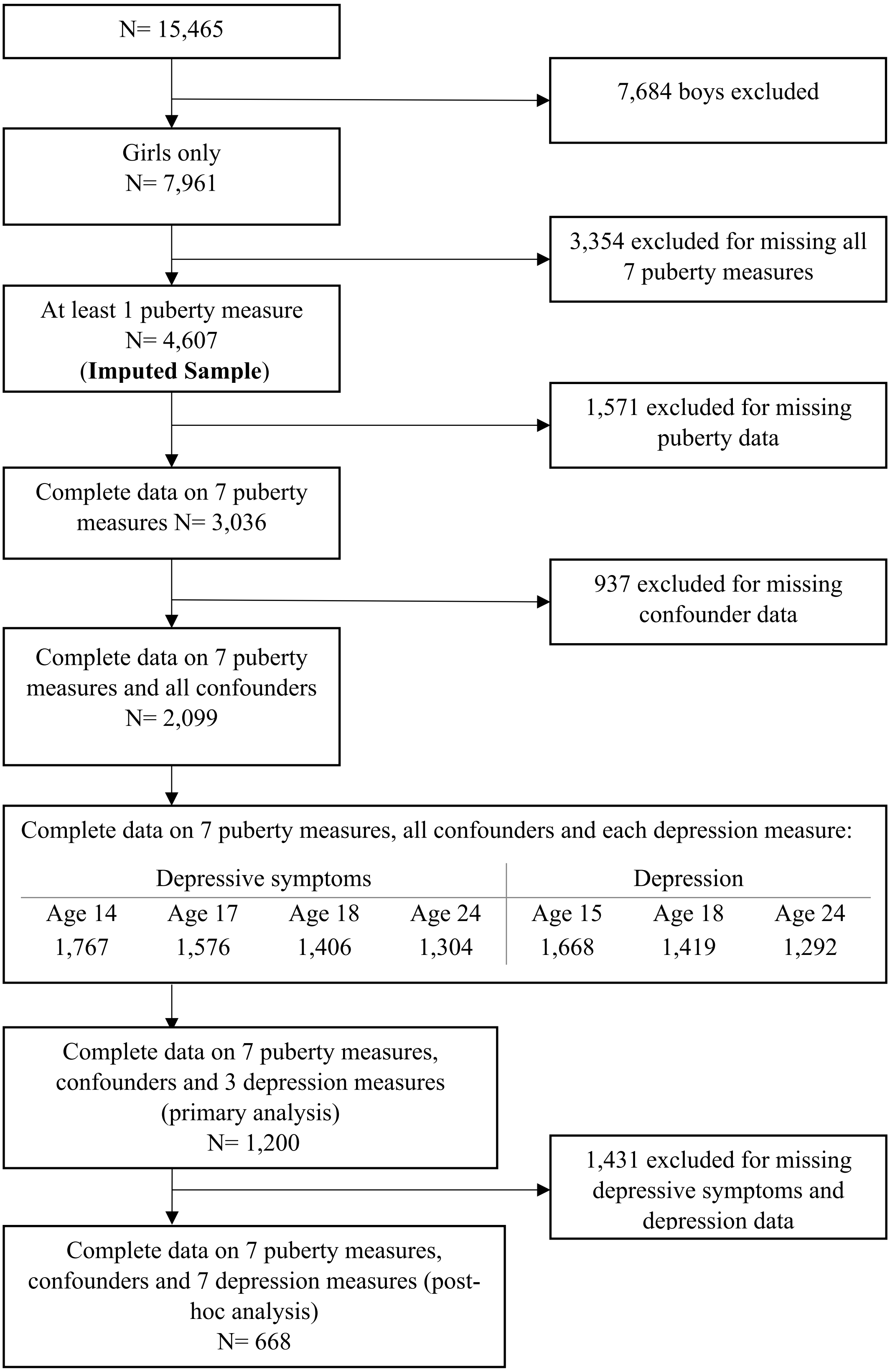

S2. Distribution of pubertal timing variables.

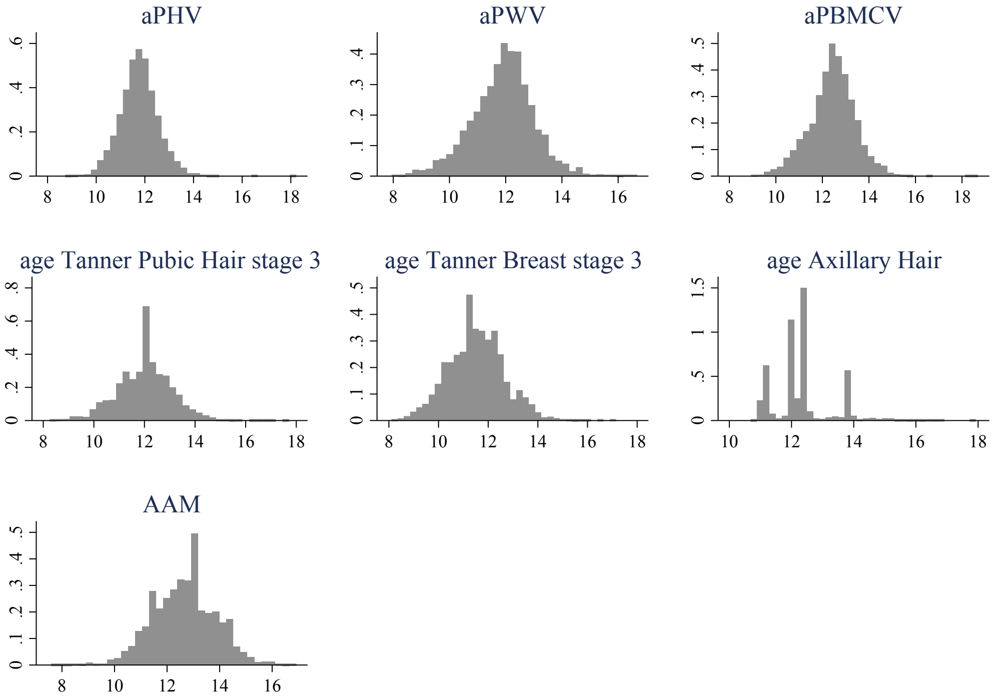

Note:
aPHV: Age at peak height velocity
aPWV: Age at peak weight velocity
aPBMCV: Age at peak bone mineral content (BMC) velocity
AAM: Age at menarche

S3. Associations between pubertal timing indicators (per one year increase) and depressive symptoms in imputed sample (N= 4,607).

	**Unadjusted**		**Adjusted for SES**		**Adjusted for SES and BMI at 9**	
	OR (95% CI)	P value	OR (95% CI)	P value	OR (95% CI)	P value
**Outcome**: Depressive symptoms (age 14)						
aPHV	0.81 (0.71, 0.91)	0.001	0.80 (0.71, 0.91)	0.001	0.82 (0.72, 0.95)	0.006
Age peak weight velocity	0.89 (0.82, 0.97)	0.007	0.88 (0.81, 0.96)	0.005	0.90 (0.82, 1.00)	0.052
Age peak BMC velocity	0.83 (0.75, 0.92)	<0.001	0.83 (0.75, 0.92)	<0.001	0.85 (0.76, 0.94)	0.003
Age Tanner pubic hair stage 3	0.88 (0.81, 0.97)	0.010	0.88 (0.80, 0.96)	0.007	0.89 (0.80, 0.98)	0.022
Age Tanner breast stage 3	0.85 (0.78, 0.93)	<0.001	0.86 (0.79, 0.94)	0.001	0.87 (0.79, 0.97)	0.009
Age axillary hair	0.91 (0.81, 1.02)	0.121	0.91 (0.81, 1.03)	0.122	0.93 (0.82, 1.04)	0.200
Age at menarche	0.82 (0.74, 0.90)	<0.001	0.83 (0.75, 0.91)	<0.001	0.84 (0.76, 0.92)	<0.001
**Outcome**: Depressive symptoms (age 17)						
aPHV	0.91 (0.82, 1.02)	0.105	0.92 (0.82, 1.03)	0.138	0.94 (0.83, 1.07)	0.373
Age peak weight velocity	0.92 (0.86, 0.99)	0.035	0.92 (0.85, 0.99)	0.024	0.93 (0.85, 1.02)	0.139
Age peak BMC velocity	0.92 (0.85, 1.00)	0.057	0.92 (0.85, 1.00)	0.050	0.94 (0.86, 1.03)	0.189
Age Tanner pubic hair stage 3	0.98 (0.90, 1.06)	0.600	0.97 (0.90, 1.06)	0.506	0.99 (0.91, 1.07)	0.758
Age Tanner breast stage 3	0.87 (0.81, 0.94)	>0.001	0.89 (0.82, 0.96)	0.002	0.90 (0.82, 0.98)	0.014
Age axillary hair	0.98 (0.89, 1.08)	0.645	0.98 (0.89, 1.07)	0.610	0.99 (0.90, 1.09)	0.788
Age at menarche	0.89	0.001	0.91	0.008	0.92	0.034

	(0.84, 0.96)		(0.85, 0.98)		(0.86, 0.99)	
**Outcome**: Depressive symptoms (age 18)						
aPHV	0.87 (0.73, 1.02)	0.089	0.89 (0.75, 1.05)	0.170	1.01 (0.84, 1.21)	0.918
Age peak weight velocity	0.88 (0.78, 0.98)	0.022	0.87 (0.78, 0.98)	0.020	0.96 (0.85, 1.09)	0.558
Age peak BMC velocity	0.86 (0.76, 0.98)	0.024	0.87 (0.76, 0.98)	0.028	0.93 (0.82, 1.07)	0.308
Age Tanner pubic hair stage 3	0.91 (0.80, 1.02)	0.118	0.92 (0.81, 1.04)	0.177	0.97 (0.85, 1.10)	0.616
Age Tanner breast stage 3	0.82 (0.73, 0.92)	0.001	0.84 (0.74, 0.94)	0.003	0.91 (0.80, 1.04)	0.172
Age axillary hair	0.88 (0.77, 1.02)	0.081	0.90 (0.76, 1.01)	0.079	0.92 (0.79, 1.06)	0.233
Age at menarche	0.91 (0.80, 1.02)	0.110	0.93 (0.82, 1.05)	0.215	1.00 (0.88, 1.14)	0.971
**Outcome**: Depressive symptoms (age 24)						
aPHV	0.95 (0.85, 1.06)	0.367	0.96 (0.86, 1.08)	0.506	1.00 (0.89, 1.12)	0.997
Age peak weight velocity	0.96 (0.88, 1.04)	0.287	0.95 (0.87, 1.04)	0.261	0.99 (0.90, 1.09)	0.840
Age peak BMC velocity	0.99 (0.90, 1.09)	0.854	0.99 (0.90, 1.09)	0.896	1.03 (0.94, 1.14)	0.521
Age Tanner pubic hair stage 3	1.04 (0.96, 1.13)	0.311	1.04 (0.96, 1.12)	0.365	1.06 (0.97, 1.15)	0.181
Age Tanner breast stage 3	0.93 (0.86, 1.00)	0.052	0.94 (0.87, 1.02)	0.149	0.97 (0.89, 1.06)	0.511
Age axillary hair	1.06 (0.96, 1.16)	0.229	1.06 (0.96, 1.16)	0.243	1.07 (0.98, 1.18)	0.144
Age at menarche	0.92 (0.84, 1.00)	0.051	0.94 (0.86, 1.03)	0.166	0.96 (0.87, 1.05)	0.353

Note: Depressive Symptoms = SMFQ>=11

SES variables include maternal education, social class, home ownership, financial problems and father absence.

S4. Associations between pubertal timing indicators (per one year increase) and depression in imputed sample (N= 4,607).

	Unadjusted		Adjusted for SES		Adjusted for SES and BMI at 9	
	OR (95% CI)	P value	OR (95% CI)	P value	OR (95% CI)	P value
**Outcome**: Depression, DAWBA measured (age 15)						
aPHV	0.82 (0.59, 1.13)	0.225	0.82 (0.59, 1.14)	0.237	0.87 (0.61, 1.25)	0.451
Age peak weight velocity	0.84 (0.69, 1.03)	0.096	0.83 (0.68, 1.01)	0.065	0.86 (0.67, 1.09)	0.209
Age peak BMC velocity	0.82 (0.64, 1.05)	0.111	0.81 (0.63, 1.04)	0.099	0.84 (0.64, 1.11)	0.225
Age Tanner pubic hair stage 3	0.79 (0.62, 1.00)	0.054	0.78 (0.61, 0.98)	0.039	0.79 (0.62, 1.01)	0.067
Age Tanner breast stage 3	0.91 (0.75, 1.19)	0.635	0.95 (0.75, 1.19)	0.635	1.01 (0.77, 1.32)	0.941
Age axillary hair	1.16 (0.91, 1.48)	0.222	1.15 (0.90, 1.47)	0.272	1.18 (0.92, 1.52)	0.189
Age at menarche	0.83 (0.66, 1.05)	0.121	0.86 (0.67, 1.09)	0.214	0.89 (0.68, 1.15)	0.358
**Outcome**: Depression, CIS-R measured (age 18)						
aPHV	0.85 (0.72, 1.00)	0.047	0.85 (0.72, 1.01)	0.059	0.86 (0.72, 1.03)	0.107
Age peak weight velocity	0.99 (0.89, 1.10)	0.864	0.99 (0.89, 1.10)	0.844	1.02 (0.90, 1.16)	0.724
Age peak BMC velocity	0.92 (0.80, 1.06)	0.273	0.92 (0.80, 1.06)	0.273	0.94 (0.80, 1.09)	0.395
Age Tanner pubic hair stage 3	0.99 (0.87, 1.12)	0.875	0.99 (0.87, 1.12)	0.838	1.00 (0.87, 1.14)	0.972
Age Tanner breast stage 3	0.83 (0.74, 0.94)	0.003	0.84 (0.75, 0.95)	0.005	0.83 (0.72, 0.95)	0.008
Age axillary hair	1.00 (0.87, 1.14)	0.977	1.00 (0.88, 1.15)	0.970	1.01 (0.88, 1.16)	0.872

Age at menarche	0.84 (0.74, 0.95)	0.005	0.85 (0.75, 0.96)	0.011	0.85 (0.75, 0.97)	0.015
**Outcome**: Depression, CIS-R measured (age 24)						
aPHV	0.81 (0.69, 0.95)	0.012	0.82 (0.70, 0.97)	0.018	0.89 (0.75, 1.06)	0.179
Age peak weight velocity	0.90 (0.81, 1.00)	0.045	0.90 (0.81, 1.00)	0.042	0.99 (0.88, 1.12)	0.919
Age peak BMC velocity	0.93 (0.82, 1.05)	0.235	0.93 (0.82, 1.06)	0.271	1.01 (0.88, 1.16)	0.854
Age Tanner pubic hair stage 3	0.97 (0.86, 1.08)	0.550	0.96 (0.86, 1.08)	0.521	1.00 (0.89, 1.13)	0.961
Age Tanner breast stage 3	0.84 (0.74, 0.96)	0.009	0.85 (0.75, 0.97)	0.015	0.91 (0.79, 1.05)	0.206
Age axillary hair	0.90 (0.78, 1.03)	0.132	0.90 (0.78, 1.03)	0.122	0.93 (0.80, 1.07)	0.279
Age at menarche	0.92 (0.82, 1.02)	0.126	0.94 (0.84, 1.05)	0.282	1.00 (0.89, 1.12)	0.953

Note: SES variables include maternal education, social class, home ownership, financial problems and father absence.

S5 Complete Case: Associations between pubertal timing indicators (per one year increase) and depressive symptoms in complete case samples

	**Unadjusted**		**Adjusted for SES**		**Adjusted for SES and BMI at 9**	
	OR (95% CI)	P value	OR (95% CI)	P value	OR (95% CI)	P value
**Outcome**: Depressive symptoms (age 14), *n = 1,767*						
aPHV	0.83 (0.71, 0.97)	0.019	0.83 (0.71, 0.97)	0.019	0.85 (0.72, 1.01)	0.070
Age peak weight velocity	0.93 (0.84, 1.04)	0.201	0.93 (0.84, 1.04)	0.202	0.97 (0.86, 1.10)	0.670
Age peak BMC velocity	0.86 (0.76, 0.98)	0.018	0.86 (0.76, 0.98)	0.018	0.88 (0.77, 1.00)	0.069
Age Tanner pubic hair stage 3	0.84 (0.75, 0.95)	0.004	0.84 (0.75, 0.95)	0.004	0.86 (0.76, 0.97)	0.013
Age Tanner breast stage 3	0.86 (0.77, 0.96)	0.009	0.86 (0.77, 0.96)	0.010	0.88 (0.77, 1.00)	0.047
Age axillary hair	0.87 (0.75, 0.99)	0.043	0.87 (0.75, 0.99)	0.042	0.88 (0.77, 1.01)	0.078
Age at menarche	0.83 (0.74, 0.93)	0.002	0.83 (0.74, 0.94)	0.002	0.85 (0.75, 0.96)	0.008
**Outcome**: Depressive symptoms (age 17), *n = 1,576*						
aPHV	0.96 (0.83, 1.11)	0.596	0.98 (0.84, 1.13)	0.742	0.99 (0.84, 1.17)	0.930
Age peak weight velocity	0.96 (0.87, 1.06)	0.426	0.96 (0.87, 1.07)	0.482	0.97 (0.86, 1.10)	0.668
Age peak BMC velocity	0.93 (0.83, 1.05)	0.229	0.93 (0.83, 1.05)	0.243	0.94 (0.83, 1.06)	0.320
Age Tanner pubic hair stage 3	0.97 (0.87, 1.08)	0.592	0.98 (0.88, 1.09)	0.686	0.99 (0.88, 1.10)	0.804
Age Tanner breast stage 3	0.89 (0.80, 0.99)	0.028	0.91 (0.82, 1.00)	0.074	0.90 (0.80, 1.02)	0.092
Age axillary hair	0.98 (0.87, 1.10)	0.703	0.98 (0.87, 1.11)	0.791	0.99 (0.88, 1.12)	0.874
Age at menarche	0.92 (0.83, 1.03)	0.145	0.94 (0.84, 1.04)	0.145	0.94 (0.84, 1.05)	0.283

**Outcome**: Depressive symptoms (age 18), *n = 1,406*						
aPHV	0.92 (0.79, 1.07)	0.300	0.95 (0.81, 1.10)	0.491	1.05 (0.89, 1.24)	0.590
Age peak weight velocity	0.94 (0.85, 1.04)	0.238	0.94 (0.85, 1.04)	0.225	1.02 (0.91, 1.14)	0.778
Age peak BMC velocity	0.91 (0.81, 1.02)	0.104	0.91 (0.81, 1.03)	0.128	0.97 (0.86, 1.10)	0.629
Age Tanner pubic hair stage 3	0.96 (0.86, 1.07)	0.456	0.97 (0.87, 1.09)	0.608	1.01 (0.90, 1.14)	0.829
Age Tanner breast stage 3	0.84 (0.76, 0.94)	0.002	0.86 (0.78, 0.96)	0.008	0.92 (0.81, 1.03)	0.153
Age axillary hair	0.94 (0.83, 1.07)	0.370	0.94 (0.83, 1.07)	0.368	0.97 (0.86, 1.10)	0.669
Age at menarche	0.93 (0.83, 1.04)	0.216	0.95 (0.85, 1.07)	0.394	1.00 (0.90, 1.14)	0.896
**Outcome**: Depressive symptoms (age 24), *n = 1,304*						
aPHV	0.92 (0.80, 1.07)	0.299	0.94 (0.81, 1.09)	0.409	0.97 (0.82, 1.13)	0.668
Age peak weight velocity	0.95 (0.86, 1.06)	0.382	0.96 (0.86, 1.06)	0.425	0.99 (0.87, 1.11)	0.812
Age peak BMC velocity	0.97 (0.87, 1.10)	0.672	0.98 (0.87, 1.10)	0.743	1.01 (0.89, 1.14)	0.934
Age Tanner pubic hair stage 3	1.01 (0.91, 1.13)	0.826	1.02 (0.91, 1.14)	0.747	1.04 (0.92, 1.16)	0.539
Age Tanner breast stage 3	0.93 (0.84, 1.04)	0.191	0.96 (0.86, 1.06)	0.420	0.98 (0.87, 1.11)	0.779
Age axillary hair	1.05 (0.93, 1.19)	0.402	1.07 (0.94, 1.21)	0.293	1.09 (0.96, 1.23)	0.202
Age at menarche	0.90 (0.80, 1.00)	0.056	0.91 (0.81, 1.02)	0.108	0.92 (0.82, 1.04)	0.195

Note: Depressive Symptoms = SMFQ>=11
SES variables include maternal education, social class, home ownership, financial problems and father absence.

S6. Complete Case: Associations between pubertal timing indicators (per one year increase) and depression in complete case sample

	Unadjusted		Adjusted for SES		Adjusted for SES and BMI at 9	
	OR (95% CI)	P value	OR (95% CI)	P value	OR (95% CI)	P value
**Outcome**: Depression – DAWBA (age 15), *n = 1,668*						
aPHV	0.68 (0.46, 1.01)	0.058	0.68 (0.46, 1.01)	0.059	0.74 (0.48, 1.14)	0.169
Age peak weight velocity	0.73 (0.57, 0.96)	0.023	0.73 (0.56, 0.95)	0.019	0.76 (0.56, 1.04)	0.087
Age peak BMC velocity	0.73 (0.55, 0.98)	0.041	0.73 (0.54, 0.98)	0.038	0.78 (0.56, 1.07)	0.128
Age Tanner pubic hair stage 3	0.74 (0.56, 1.00)	0.046	0.74 (0.56, 0.99)	0.042	0.77 (0.58, 1.04)	0.090
Age Tanner breast stage 3	0.82 (0.62, 1.09)	0.171	0.84 (0.64, 1.11)	0.232	0.92 (0.68, 1.27)	0.625
Age axillary hair	1.09 (0.81, 1.47)	0.577	1.08 (0.80, 1.46)	0.622	1.13 (0.83, 1.53)	0.434
Age at menarche	0.73 (0.55, 0.97)	0.032	0.74 (0.56, 0.99)	0.041	0.78 (0.58, 1.05)	0.106
**Outcome**: Depression – CIS-R (age 18), *n = 1,419*						
aPHV	0.87 (0.69, 1.08)	0.202	0.87 (0.69, 1.09)	0.236	0.89 (0.69, 1.14)	0.344
Age peak weight velocity	0.93 (0.80, 1.08)	0.341	0.92 (0.79, 1.07)	0.299	0.94 (0.79, 1.11)	0.463
Age peak BMC velocity	0.85 (0.72, 1.00)	0.062	0.84 (0.71, 1.00)	0.053	0.85 (0.71, 1.02)	0.082
Age Tanner pubic hair stage 3	0.91 (0.77, 1.07)	0.250	0.91 (0.77, 1.08)	0.271	0.92 (0.78, 1.09)	0.349
Age Tanner breast stage 3	0.77 (0.65, 0.90)	0.001	0.78 (0.66, 0.91)	0.002	0.75 (0.63, 0.90)	0.002
Age axillary hair	0.94 (0.78, 1.14)	0.544	0.94 (0.78, 1.14)	0.530	0.95 (0.79, 1.15)	0.606
Age at menarche	0.84 (0.71, 0.99)	0.038	0.85 (0.72, 1.00)	0.046	0.85 (0.72, 1.01)	0.067

**Outcome**: Depression – CIS-R (age 24), *n = 1,292*						
aPHV	0.83 (0.67, 1.02)	0.082	0.84 (0.68, 1.04)	0.103	0.92 (0.74, 1.15)	0.479
Age peak weight velocity	0.93 (0.81, 1.07)	0.320	0.94 (0.81, 1.08)	0.361	1.04 (0.89, 1.23)	0.612
Age peak BMC velocity	0.94 (0.80, 1.11)	0.501	0.95 (0.81, 1.12)	0.555	1.04 (0.87, 1.25)	0.651
Age Tanner pubic hair stage 3	0.93 (0.80, 1.08)	0.327	0.93 (0.80, 1.08)	0.352	0.97 (0.83, 1.13)	0.745
Age Tanner breast stage 3	0.83 (0.71, 0.96)	0.011	0.83 (0.72, 0.97)	0.017	0.89 (0.75, 1.05)	0.178
Age axillary hair	0.90 (0.75, 1.08)	0.252	0.90 (0.75, 1.08)	0.255	0.94 (0.78, 1.12)	0.477
Age at menarche	0.93 (0.80, 1.08)	0.353	0.94 (0.81, 1.10)	0.450	1.01 (0.86, 1.19)	0.883

Note: SES variables include maternal education, social class, home ownership, financial problems and father absence.

S7. Coding of Variables

Variables	Description and source of data collection	Data type
Pubertal timing variables		
Age at peak height velocity (aPHV)	Derived from nine repeated measures of height (to nearest 0.1cm) using stadiometer from mean age 7.6 to 17.8 by accredited fieldworkers.	Continuous – Age (in years)
Age at peak weight velocity (aPWV)	Derived from nine repeated measures of weight (to nearest 0.1kg) using Tanita Body Fat Analyser from mean age 7.6 to 17.8 by accredited fieldworkers.	
Age at peak BMC velocity (aPBMCV)	Derived from nine repeated measures of total body (less head) bone mineral content (BMC; in grams) using Lunar Prodigy Dual-energy X-ray Absorptiometry (DXA) scans from mean age 9.9 to 17.8 by accredited fieldworkers.	
Age in Tanner stage 3 of pubic hair development	Derived from nine puberty questionnaires reported by parent and/or child from mean age 8.2 to 17.0 years, using line drawings with accompanying descriptions of the five Tanner stages of pubic hair development to identify the stage most closely matched with the child's current stage.	
Age in Tanner stage 3 of genitalia development	Derived from nine puberty questionnaires reported by parent and/or child from mean age 8.2 to 17.0 years, using line drawings with accompanying descriptions of the five Tanner stages of genitalia development to identify the stage most closely matched with the child's current stage.	
Age at voice break	Derived from eight puberty questionnaires reported by parent and/or child from mean age 9.7 to 17.0 years, asking whether the participant's voice (1) had not changed, (2) was occasionally a lot lower, or (3) had changed totally.	
Age at axillary hair	Derived from seven puberty questionnaires by parent and/or child from mean age 9.7 to 17.0 years, asking whether the participant had (1) started growing hair in the armpits or (2) not yet started.	
Depression Variables		
Depressive Symptoms (SMFQ)	Measured at 13.84, 16.67, 17.84 and 23.87 years using Short Moods and Feeling Questionnaire (SMFQ)	Continuous variable: ranging from 0-26<br>Categorical Variable: Cut-off point at 11

		0: No depressive symptoms; 1: Depressive symptoms
Depression (DAWBA)	Measured at 15.49 years using the developmental and wellbeing assessment (DAWBA) band predictions.	Ordered categorical variable: 1 = <0.1%, 2 = ~0.5%, 3 = ~15%, 4 = ~50%, 5 = >70%<br>Binary categorical: cut off-point at >50%<br>0 = No depression; 1 = Depression
Depression (CIS-R)	Measured at 17.82 and 24.45 years using Revised Clinical Interview Schedule (CIS-R), according to ICD-10 Diagnosis of depression (any severity: mild, moderate or severe)	Categorical Variable:<br>0: No depression; 1: Depression
Confounding Variables		
Home ownership	Mother completed; sent when child was 1y9m<br>" Is your home: Being bought/mortgaged = 0, Being bought from council = 1, Owned with no mortgage = 2, Rented from council = 3, Rented from private landlord (furnished) = 4, Rented from private landlord (furnished) = 5, Rented from housing association = 6"	Categorical Variable:<br>0 - House rented from council/housing association/bought from council (1,3,6)<br>1 - House privately rented/ owned/mortgaged (0,2,4,5)
Maternal Education	Mother completed; sent when child was 5y1m<br>"Mother has/is: No educations quals, CSE/GCSE, Vocational qual, Apprenticeship, State enrolled nurse, City & Guilds Intermediate Technical Quals, City & Guilds Final Technical Quals, City & Guilds Full Technical Quals, O-Level/GCSE, A Levels, Teaching qual, University degree, State Registered Nurse."	Ordered categorical Variable<br>0 = < O Level<br>1 = O Level<br>2 = > O Level
Major Financial problems	Mother completed; sent when child was <1, 2 and 5 years old.	Categorical Variable<br>0 - No financial Problems<br>1 - Financial Problems
Social Class	Mother completed; sent prior to birth. Reported both maternal and partner social class: "I =1, II = 2, III (Non-Manual) = 3, III (Manual) = 4, IV = 5, V = 6"	Categorical variable

		0 Non-Manual (1, 2, 3, 4)<br>1 Manual (5, 6)
Father absence	Recorded by mother at various timepoints<br>"How old was the child when the natural father stopped living with the child?"	Ordered categorical variable<br><br>0 Father present<br>1 Father left before 5 years<br>2 Father left between 5-10 years
BMI at 9	Calculated at age 9 based on height and weight measurements obtained from clinics and questionnaire data when clinic data was missing.	Continuous Measure
Auxiliary Variables		
BMI at 7	Calculated at age 9 based on height and weight measurements obtained from clinics and questionnaire data when clinic data was missing.	Continuous variable
Crowding Index	Measured at 8 weeks gestation<br>Derived: Number of people in the household divided by number of rooms	Ordered categorical variable<br>1 <=0.5<br>2 >0.5 – 0.75<br>3 >0.75 -1<br>4 > 1
Car Access	Measured at 8 weeks gestation<br>"Do you or your partner have the use of a car (including vans, minibuses, etc.): Yes/No?"	Categorical variable<br>0 No car access<br>1 Car access
Weekly Income	Measured when child was 47 months<br>"On average, how much is the take home family income each week (include social benefits etc.)?: 1 < £100, 2 £100 - £199, 3 £200 - £299, 4 £300- £399, 5 > £400"	Ordered categorical variable<br>1 < £100<br>2 £100 - £199<br>3 £200 - > £400<br>4 £300- £399<br>5 > £400
Depressive Symptoms at age 13	Short Moods and Feeling Questionnaire (SMFQ)<br>Measured at 13 years	Continuous variable ranging from 0-26

Strengths and Difficulties at age 12	Strength and Difficulties<br>Derived Variable: Continuous score (Range 0-40)<br>kw6602a – Emotional symptoms score	Categorical variable
Maternal depression	Edinburgh Postnatal depression scale	Continuous Variable
